# Supplementary figures and images for: GLUT4 expression and glucose transport in human induced pluripotent stem cell-derived cardiomyocytes
Source: PLoS One. 2019 Jul 25;14(7):e0217885. doi: 10.1371/journal.pone.0217885 (PMC6657831; doi:10.1371/journal.pone.0217885)

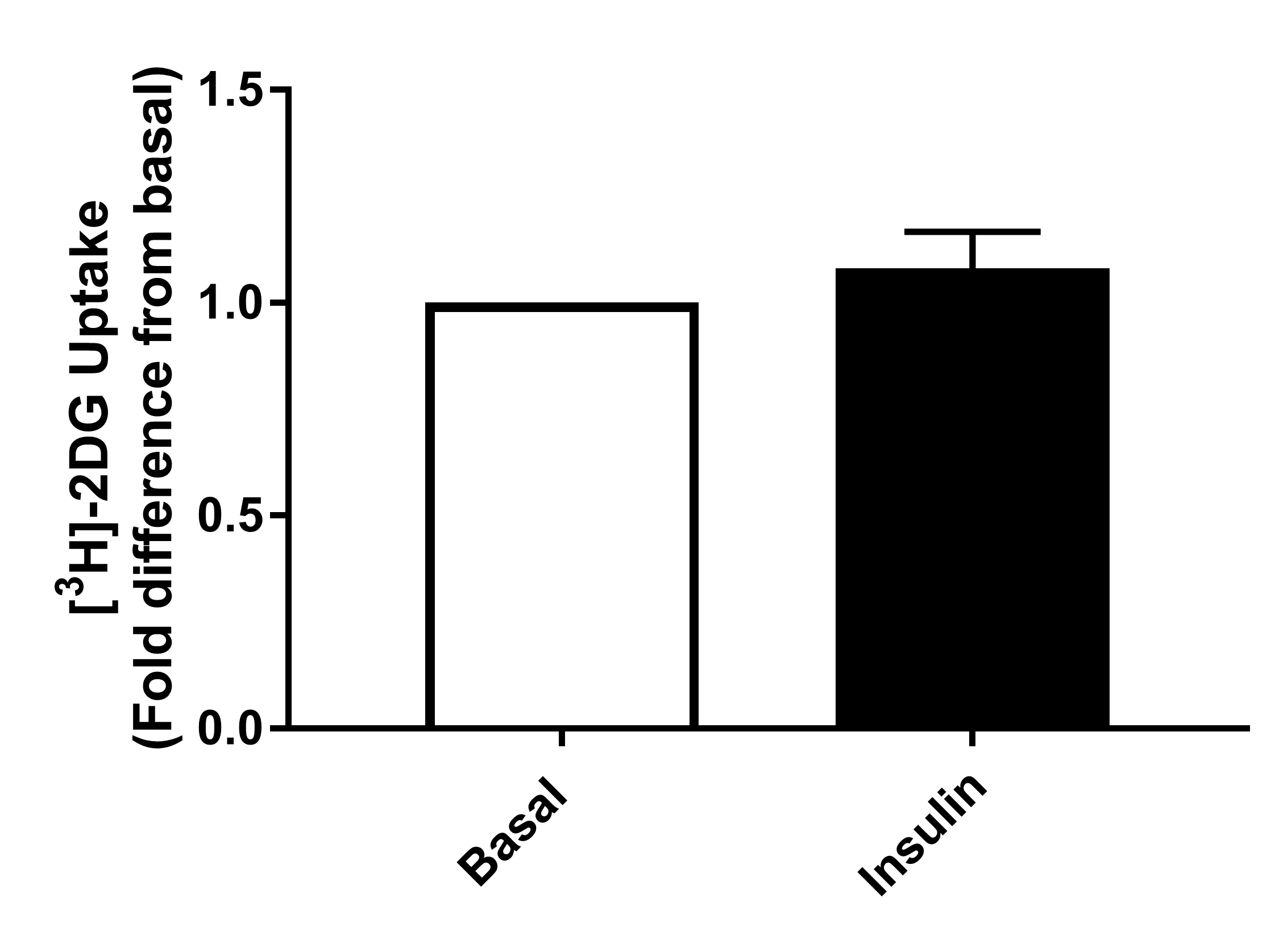

Supplement: S1 Fig — Background corrected [3H]-2-deoxyglucose uptake was recorded from iPSC-CM, via the protocol detailed in the methods. Cells were insulin stimulated for 30 min prior to incubation with [3H]-2-deoxyglucose assay mix for 15 min. Data is displayed as the mean (+S.E.M.) fold change in uptake relative to basal values from 3 representative individual experiments. Statistical testing was performed with a 2-way ANOVA on raw unadjusted data, and the level of significance was set at P = 0.05. (TIF) [file pone.0217885.s001.tif]

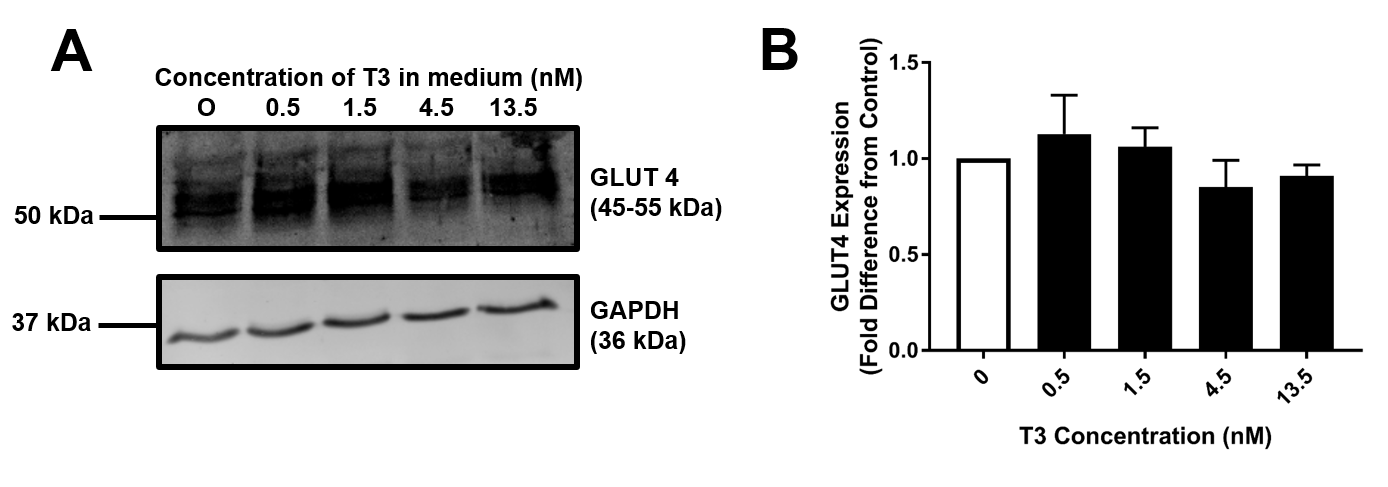

Supplement: S2 Fig — iPSC-CM were maintained for 4 days in medium containing 0–13.5 nM T3 as indicated. Subsequently, protein lysates were generated and subjected to SDS-PAGE and immunoblotting. Lysates were incubated with antibodies probing for GLUT4 (1:2000, 1% milk, PBS-T) and GAPDH (1:80,000, 1% milk, PBS-T). Quantification was performed via densitometry and the mean (+S.E.M.) expression of GLUT4 (normalised to GAPDH) relative to control for each condition across 3 independent experiments is displayed. Statistical analysis was performed on unadjusted data with a 1-way ANOVA, and the level of significance was set at P = 0.05. (TIF) [file pone.0217885.s002.tif]
